# Prevalence of diabetes among homeless and slum dwellers in Accra, Ghana: a survey study

**DOI:** 10.1186/s13104-019-4613-5

**Published:** 2019-09-11

**Authors:** Ahmed Tijani Bawah, Albert Abaka-Yawson, Mohammed Mustapha Seini, Francis Agyemang Yeboah, Robert Amadu Ngala

**Affiliations:** 1 Department of Medical Laboratory Sciences, School of Allied Health Sciences, University of Health and Allied Health Sciences, Ho, Ghana; 2 Greater Accra Regional Hospital, Accra, Ghana; 3 Department of Molecular Medicine, School of Medical Sciences, Kwame Nkrumah University of Science and Technology, Kumasi, Ghana

**Keywords:** Homeless, Diabetes mellitus, Hemoglobin A1c, Head porters

## Abstract

**Objective:**

This study aimed at determining diabetes status of homeless people in Nima and Agbogbloshie, Accra, Ghana and to evaluate the association between socio-demographic characteristics and diabetes prevalence.

**Results:**

A total of 130 homeless and slum dwellers took part in the study out of which 7 (5.4%) participants were diagnosed with diabetes while 13 (10%) were considered as having prediabetes. This is slightly lower than what had been reported two decades ago but similar to overall estimates of diabetes prevalence in Africa (5.7%). Diagnosis of normoglycemia, prediabetes and diabetes was based on individual’s hemoglobin A1c (HbA1c) level: ≤ 5.9%, 6.4–6.0%, and ≥ 6.5%, respectively. There was no significant association between prevalence of diabetes or prediabetes and the socio-demographic characteristics of the participants. The slightly lower diabetes prevalence among the homeless and slum dwellers compared to the general population may be due to constant movement of these people in the streets, a practice that could serve as a form of exercise for them. Intensive social support aimed at preventing and managing diabetes is crucial if we are to further reduce the incidence of diabetes in homeless people.

## Introduction

Diabetes mellitus is one of the commonest non-communicable diseases worldwide and its prevalence is increasing in both developed and developing countries [1] as a result of urbanization and dramatic changes in life styles. Lack of access to health care systems and essential medication, late presentation and late diagnosis coupled with poor management of diabetes have created substantial burden on the socioeconomic conditions for most diabetes patients in developing countries [2].

Treatment of diabetes mellitus require multidisciplinary approach, however, most homeless Ghanaians including female head porters (*Kayayei*) experience difficulties in accessing health care services in Accra as a result financial challenges [3]. Furthermore, linguistic differences between health professionals and patients restrict most of these homeless migrants from seeking healthcare services from accredited health facilities and some of them resort to patronizing the services of informal care providers, though a few of them may be insured under the National Health Insurance Scheme (NHIS) [3]. Diabetic complications may exhibit sudden onset due to lack of subjective symptoms, therefore accurate and reliable data on the prevalence of diabetes among homeless people in Ghana will help policy makers in creating awareness and support services for this vulnerable group of our society.

Prevalence of diabetes in homeless people in Sub-Saharan African countries including Ghana is not known, however, few studies in Japan [4], Ireland [5], United States of America [6] and France [7] have provided some information on the prevalence of the disease in these vulnerable members of the population. To this end, this study aimed at determining the prevalence of diabetes among homeless and slum dwellers in two suburbs (Agbogbloshie and Nima) of Accra, Ghana and examined the association between diabetes and socio-demographic background of participants.

## Main text

### Methods

The participants in this study were selected using convenience-sampling procedure because majority of them were porters with no permanent places of residence and their activity highly mobile, a situation which called for the use of nonprobability sampling method [8]. Inclusion in this study was based on the United Nations definition of homelessness which states that an individual is homeless if he/she has no stable home, living either in a temporary shelter or unsheltered location not meant for human inhabitance (e.g., parked car, kiosk, the street, or a subway station) [9].

A total 130 of homeless subjects took part in the study. Information on participants’ characteristics such as age, gender, residence status, ethnicity, educational level and duration of homelessness was recorded. Weight and height were measured and body mass index (BMI) calculated using the formula: bodyweight (kg)/[height (m)]^2^. The participants were grouped into underweight (BMI < 18.5), normal (BMI 18.5–24.9 kg/m^2^), overweight (BMI = 25–29.9 kg/m^2^) and obese (BMI ≥ 30 kg/m^2^). Five milliliters of fasting blood sample was taken into fluoride tubes and ethylendiamine tetra acetic tubes.

#### Biochemical analysis and outcome measurement

The hematocrit and hemoglobin were estimated using SYSMEX XS–500i auto-hematology analyzer (Sysmex Europe GmbH, Bornbarch1 22848 Norderstedt, Germany). Hemoglobin A1c (HbA1c) was determined using high performance liquid chromatography (Tosoh G7; Tosoh Corporation, Tokyo, Japan) while fasting blood glucose was determined using Selectra Pro S (Vital Scientific B.V. Van Rensselaerwweg 4, NL 6956 AV Spankeren, The Netherlands) automated chemistry analyzer. Serum HbA1c measurement was used to screen for diabetes mellitus instead of 75 g oral glucose tolerance test (OGTT) because the study participants were highly mobile and also getting them to adhere to instructions for the OGTT before and during the procedure [10] was not possible. Those whose HbA1c levels were < 5.9% were diagnosed as normoglycemia while those with HbA1c value of 6.0–6.4% and ≥ 6.5% were diagnosed as prediabetes and diabetes respectively.

#### Statistical analysis

The data was first entered into Microsoft Office Excel 2007 and Graph Pad Prism 3.02 software was used to analyze the data. The level of statistical significance was set at p < 0.05 for all tests and at 95% confidence interval. Proportions of those with diabetes, prediabetes and normoglycemia were calculated and Chi square (χ^2^) tests used to analyze the associations between the different parameters.

### Results

The participants in this study comprised 115 (88.5%) homeless Ghanaians and 15 (11.5%) non Ghanaian homeless people. Among the Ghanaians, 91 (70%) originated from the northern regions of Ghana and the remaining 24 (18.5%) originated from other parts of the country. Majority of the participants lived in the streets, kiosks, shacks or other temporary structures in slums around Nima and Agbogbloshie in Accra for periods ranging from < 1 year (28.5%) to ≥ 5 years (6.9%). Most of the participants who hailed from the Northern parts of the country were head porters popularly referred to as Kayayei (plural), Kayayo (singular).

Alcohol consumption (3.1%, n = 4) and smoking (0.8%, n = 1) status were very low among participants and most of them (40.0%, n = 52) had no formal education. Those with formal education were primary (23.8%, n = 31), Middle/Junior high school (24.6%, n = 32), Vocational/technical/ senior high school/ ordinary and advanced level (11.5%, n=15). A total of 34 (26.1%) were obese, 43 (33.1%) overweight, 49 (37.7%) had normal weight and 4 (3.1%) were underweight (Table 1).

**Table 1:** Socio-demographic characteristics of the participants

Gender	Male n (%)	Female n (%)	Total (%)
	59 (45.4)	71 (54.6)	130 (100)
Age (years)			
20–29	15	27	42 (32.3)
30–39	20	29	49 (37.7)
40–49	14	11	25 (19.2)
50–59	6	3	9 (6.9)
60–69	3	1	4 (3.1)
> 70	1	0	1 (0.8)
BMI classification			
Underweight	1	3	4 (3.1)
Normal weight	30	19	49 (37.7)
Overweight	15	28	43(33.1)
Obese	13	21	34 (26.1)
Duration of homelessness (years)			
≤ 1	9	28	37 (28.5)
2	23	29	52 (40.0)
3	15	10	25 (19.2)
4	5	2	7 (5.4)
≥ 5	7	2	9(6.9)
Alcohol consumption			
Yes	3	1	4 (3.1)
No	56	70	126 (96.9)
Smoking			
Yes	1	0	1 (0.8)
No	58	71	129 (99.2)
Education level			
No education	20	32	52 (40.0)
Primary	17	14	31 (23.8)
Middle/JHS	18	14	32 (24.6)
Voc/Tech/SHS/O’/A’ Level	4	11	15 (11.5)

None of the participants had severe anemia and so the effect of low hemoglobin on the HbA1c was ruled out. The prevalence of prediabetes and diabetes in the 130 homeless and slum dwellers were 10% (110) and 5.4% (7) respectively. The prevalence of diabetes increased from 0% in those who were underweight (BMI ≤ 18.5 kg/m^2^) to 8.8% in the obese respondents (BMI ≥ 30 kg/m^2^). The prevalence of diabetes was 2.4%, 6.1%, 8%, and 11.1% in those aged 20–29, 30–39, 40–49 and 50–59 years respectively (Table 2). None of those aged 60–69 and above 70 years screened positive for diabetes. This is because; homeless persons with diabetes mellitus might die from diabetic complications before they reach advanced age as a result of lack of access to medical care. There was no discrepancy between blood glucose and the HbA1c levels though we did not use 75 g glucose tolerance test to diagnose the participants’ with diabetes mellitus.

**Table 2:** Prevalence of HbA1c stratified by BMI category and generation

	HbA1c (%)			Total
	< 5.9 n (%)	6.0–6.4 n (%)	≥ 6.5 n (%)	
Age (years)				
20–29	41 (97)	0 (–)	1 (2.4)	42
30–39	41 (83.7)	5 (10.2)	3 (6.1)	49
40–49	17 (68)	6 (24)	2 (8)	25
50–59	7 (77.8)	1 (11.1)	1 (11.1)	9
60–69	3 (75)	1 (25)	0 (–)	4
≥ 70	1 (100)	0 (–)	0 (–)	1
Total	110 (84.6)	13 (10)	7 (5.4)	130
BMI (kg/m^2^)				
Underweight	4 (100)	0 (–)	0 (–)	4
Normal weight	44 (89.8)	4 (8.2)	1 (2)	49
Overweight	36 (83.7)	4 (9.3)	3 (7)	43
Obese	26 (76.5)	5 (14.7)	3 (8.8)	34
Total	110 (84.6)	13 (10)	7 (5.4)	130
Blood glucose (mmol/L)				
Mean ± SD	5.07 ±0.514	6.495 ±0.268	11.38 ±3.898	
Min–max	3.9–6.0	6.1–6.900	7.5–18.78	

HbA1c = Glycated hemoglobin, BMI = body mass index, BMI of <18.5 = underweight, 18.5–24.9 = normal weight, 25–29.9 = overweight, ≥ 30 = Obese

Analysis of the prevalence of diabetes stratified by background of participants revealed significant association (p < 0.0001) between alcohol consumption and diabetes mellitus with 25% of those who consume alcohol screening positive for diabetes mellitus as against with 4.8% of non-alcohol consumers. Other factors such us; smoking, duration of homelessness, educational level, ethnic background and gender of participants did not show any significant associations (Table 3).

**Table 3:** Prevalence of diabetes stratified by background according to HbA1c levels

Background of participants	Total n (%)	HbA1c n (%)		
		≤ 5.9	6.0–6.4	≥ 6.5
Total	130 (100)	110 (84.6)	13 (10)	7 (5.4)
Gender				
Male	59	51(86)	5 (8.5)	3 (5.1)
Female	71	59 (83)	8 (11.3)	4 (5.6)
Duration of homelessness (years)				
≤ 1	37	32 (86.5)	3 (8.1)	2 (7.4)
2	52	42 (80.8)	7 (13.5)	3 (5.8)
≥ 3	41	36 (80.8)	3 (7.3)	2 (4.9)
Alcohol consumption				
Yes	4	2 (50.0)	1 (25)	1(25)*
No	126	108 (85.7)	12 (9.5)	6 (4.8)*
Smoking				
Yes	1	1 (100.0)	0 (–)	0 (–)
No	129	109 (84.5)	13 (10.1)	7 (5.4)
Education level				
No education	47	38 (80.9)	6 (12.8)	3 (6.3)
Primary	29	24 (82.8)	3 (10.3)	2 (6.9)
Middle/JHS	31	27 (87.1)	3 (9.7)	1(3.2)
Voc/Tech/SHS/O’/A’ Level	21	19 (90.5)	1(4.8)	1(4.8)
Tertiary	2	2 (100.0)	0 (–)	0 (–)
Ethnic background				
Northerners	91	80 (87.9)	8 (8.8)	3 (3.3)
Other Ghanaians	24	18 (75.0)	4 (16.7)	2 (8.3)
Non-Ghanaians	15	12 (80.0)	1 (6.7)	2 (13.3)

*p < 0.0001

### Discussion

This study was aimed at determining the prevalence of diabetes among homeless Ghanaians. Diabetes mellitus is the commonest chronic disease in most countries and its prevalence continue to increase due to increasing urbanization resulting in the consumption of high amount of processed foods, lack of exercise and increasing prevalence of overweight and obesity. The rural urban migration may also lead to reduced physical activity and increased obesity which are both risk factors of diabetes mellitus. The burden of diabetes in all countries, particularly in low and middle income countries where resources are limited, continue to increase and this could be same for homeless people in most of the slums in the big cities in Africa and other parts of the world. People living with diabetes mellitus are projected to increase globally in both developed and developing countries [11].

There is very little information on the prevalence of diabetes in homeless people in Ghana hence this study was conducted and also in the light of the fact that the phenomenon of homelessness has assumed socio-medical issue with high morbidity and mortality among this vulnerable group of the population even in the developed world [12]. Most of the homeless people migrated from the northern parts of the country with few coming from the rest of the country and other neighboring countries. Aside living in dilapidated structures, these homeless people do not have access to basic services with about 92% of those in Agbogbloshie and 60% of those in Nima having no access to portable drinking water [12], consequently homelessness is an independent predictor of death from specific illnesses [13].

Our study showed that 5.4% and 10% of homeless migrants living in slums around Nima and Agbogbloshie in Accra, Ghana, had diabetes and prediabetes mellitus respectively. This is slightly lower than the 6.3% which was reported almost two decades ago [14] but it is in consonance with IDF’s global estimates of diabetes prevalence and projections from 2013 to 2035 [1]. A survey in Ireland showed prevalence of diabetes among homeless Irish population to be 8% whilst prediabetes was found to be 10% [5]. Of the 5.4% (7 out of 130) participants in this present study with DM, 42.9% were men (n = 3) and 57.1% (n = 4) were women. The percentage of men and women with diabetes were; 5.1% and 5.6% respectively while 8.5% and 11.3% of men and women respectively, had prediabetes. This is contrary to what had been reported in Ireland, where among the 8% of homeless people with diabetes, 85% of them were men and 15% were women [5]. In another study in Japan, the prevalence of diabetes in homeless people was reported to be 6.6% [4]. Differences in the population dynamics, diet and eating habits could be responsible for the differences in the prevalence of diabetes in homeless populations in different parts of the world. Furthermore, there are differences in the prevalence of diabetes among the general population of different countries. Our study also revealed higher prevalence of diabetes among obese homeless population which is in consonance with the study of prevalence of diabetes, prediabetes and metabolic syndrome among Irish homeless population [5]. A previous study in Nagoya, Japan reported 9.4% prevalence of diabetes among homeless people who consume alcohol as compared to 2.4% of their counterparts who did not consume alcohol [4] and in line with this present study which showed association between alcohol consumption and diabetes mellitus among homeless people in Nima and Agbogbloshie, Accra.

The prevalence of diabetes in this study is in line with existing data on diabetes prevalence in Ghana, therefore more attention needs to be directed to this group of people since they may be reluctant to visit hospital because of financial problems and may not be diagnosed early. It has been estimated that in Ghana, about 70% of cases of diabetes are underdiagnosed as a result of lack of awareness a situation that could lead to late presentation of the disease with its accompanying complications [14] and in Tanzania 80% to 90% people with diabetes have not been diagnosed [15]. Patients with diabetes mellitus will require regular visits to health care facilities for medical care which could be very expensive for individuals, families, society and health professionals and could have effects on national productivity [16]. The national health insurance scheme (NHIS) in Ghana has been helpful in reducing the cost of health care in the country, however, the country is still struggling to achieve NHIS’s goal of universal health care because many members do not renew their insurance policies annually [17], especially the homeless men and women including the “*Kayayei”* who cannot afford to renew their policies because of financial constraints and lack of awareness of existence of exemption policies if any.

Although the prevalence of diabetes in our cohort was slightly lower than that of the background population (6.3%), this study shows that diabetes is still a major chronic health problem among this vulnerable group. The daily struggles of these people for livelihood, lack of NHIS cards, as well as other social factors make health needs of these people a distant priority, hence they are most likely to present themselves for treatment during the later stages of diabetes with complications such as heart diseases, retinopathy, nephropathy and neuropathy which are 3.5 times higher in those with lower socioeconomic status [18].

### Conclusions

The incidence of diabetes among homeless Ghanaians was slightly lower than the general population which could be due to constant movement of these people in the streets, a practice that could serve as a form of exercise for them. However, homelessness affects a great number of Ghanaians especially migrants from the north to the slums in the cities and is associated with poverty and diseases. Many of these homeless people seek medical care from drug stores and road-side herbalists in the lorry stations, a situation which could make early diagnosis and management of diabetes difficult.

## Limitation

The estimation of the prevalence of diabetes was based on single measurements of HbA1c and fasting blood glucose which might lead to over or under estimation of diabetes prevalence.

## Data Availability

The data are available from the corresponding author on reasonable request.

## Abbreviations

BMI: body mass index
HbA1c: hemoglobin A1c
